# Trapidil attenuates diabetic cardiomyopathy via GPX3/Nrf2-mediated inhibition of myocardial pyroptosis

**DOI:** 10.3389/fphar.2025.1566622

**Published:** 2025-06-03

**Authors:** Zihao Wang, Yingzi Sun, Juanjuan Wang, Qiuyue Xu, Liuxing Wang, Qi Zhang, Juan Song, Yuchun Wang, Zhanpeng Qi

**Affiliations:** ^1^ College of Pharmacy, Qiqihar Medical University, Qiqihar, Heilongjiang, China; ^2^ College of Nursing, Qiqihar Medical University, Qiqihar, Heilongjiang, China

**Keywords:** diabetic cardiomyopathy, pyroptosis, trapidil, GPX3, Nrf2

## Abstract

**Background:**

Currently, there is a paucity of clinically effective medications for the treatment of diabetic cardiomyopathy (DCM), while the strategy of drug repurposing offers a promising avenue for advancing therapeutic development.

**Methods:**

The investigation explored the ameliorative effects and uncovered underlying mechanisms of trapidil (TRA), a drug commonly employed in the management of coronary heart disease, on DCM by inhibiting myocardial pyroptosis. Type 1 DCM models were established utilizing C57BL/6 mice and primary neonatal mouse cardiomyocytes (NMCMs), which were subsequently treated with TRA.

**Results:**

Results demonstrated that in DCM mice, TRA significantly enhanced cardiac function, effectively alleviated pathological changes in myocardial tissue, reversed ultrastructural alterations, and reduced pyroptosome formation in myocardial cells. TRA significantly increased the body weight of the mice in the DCM model group, whereas there was no significant alteration in blood glucose levels following TRA treatment. In the myocardial tissue of DCM mice and high-glucose (HG)-treated NMCMs, TRA was found to correct the aberrant expression of key proteins involved in pyroptosis, including cleaved-caspase1, NLRP3, phospho-NF-κB cyclooxygenase-2, interleukin Cleaved-IL-1β, Cleaved-IL-18, and gasdermin D. Furthermore, TRA effectively curtailed the excessive production of ROS and augmented the mitochondrial membrane potential in NMCMs under the HG environment. Proteomics analysis identified 90 differentially expressed proteins between DCM mice and TRA-treated mice, with glutathione peroxidase 3 (GPX3) emerging as a standout due to its critical role in the cellular antioxidant defense system. Further investigations revealed that the protein and mRNA levels of GPX3, as well as the activated Nrf2 protein levels, were significantly downregulated in the myocardial tissue of DCM mice and HG-treated NMCMs cells. However, these levels were notably upregulated following TRA treatment. Upon knocking down GPX3 mRNA expression using siRNA technology, the anti-pyroptotic effect of TRA in cardiomyocytes was markedly diminished, and the level of activated Nrf2 protein also significantly decreased.

**Conclusion:**

In conclusion, TRA holds potential for improving DCM, with the inhibition of myocardial pyroptosis via the GPX3/Nrf2 pathway playing a pivotal role. HG-induced Downregulation of the GPX3/Nrf2 pathway is a critical mechanism underlying pyroptosis in DCM. This pathway can be targeted for the design of DCM-related therapeutics, utilizing the aforementioned signaling mechanisms.

## 1 Introduction

Diabetes mellitus (DM), a prevalent metabolic ailment, is a global health issue with approximately 463 million cases reported in 2019, and it is alarmingly estimated to soar to 700 million individuals by 2045 (Harcourt et al., 2013; Saeedi et al., 2019). Diabetic cardiomyopathy (DCM), one of the most severe complication of DM, accounts for over 50% of diabetic-related fatalities. It poses a grave threat to human life and health by potentially leading to heart failure, sudden cardiac death, and other life-threatening conditions (Dillmann, 2019). Unfortunately, the pathogenesis of DCM remains intricate and elusive. The current treatment strategies for DCM, which primarily focus on diet control, blood sugar management, and symptomatic relief, have demonstrated only limited efficacy (Paolillo et al., 2019). This is particularly concerning given the striking lack of truly effective therapeutic medications, leaving patients with DCM in a suboptimal treatment predicament.

DCM is characterized by structural or functional abnormalities of the heart in diabetic patients who are free from cardiovascular comorbidities. A growing body of evidence indicates that inflammation in the myocardium is a crucial element during DCM progression. Pyroptosis, a newly-recognized programmed cell death form, makes a substantial contribution to the inflammation in the myocardium during DCM (Lu et al., 2022). In DM, hyperglycemia disrupts metabolic processes, leading to mitochondrial dysfunction and damage to the antioxidant system, thereby initiating oxidative stress. Reactive oxygen species (ROS) can directly trigger the inflammasome, particularly nucleotide-binding oligomerization domain-like receptor protein 3 (NLRP3), or indirectly via Nuclear factor kappa-B (NF-κB) pathways. This activation of the NLRP3 inflammasome causes the activation of caspase-1. Caspase-1 then cleaves the precursors of IL-1β and IL-18 into their active forms and also cleaves gasdermin D (GSDMD) into N- and C-terminal fragments. The GSDMD N-terminus targets the cell membrane, binds to phospholipid proteins, polymerizes, and forms pores. Pro-inflammatory cytokines like IL-1β and IL-18 are secreted through these pores, damaging the cell membrane, releasing cell contents, and causing cell death (Fu and Wu, 2023; Wang et al., 2021). Pyroptosis exacerbates inflammation in DCM, inflicting damage on cardiomyocytes and accelerating the progression of the disease (Liu et al., 2024; Peng et al., 2022). Consequently, pyroptosis has increasingly been recognized as a critical therapeutic target for the management of DCM (Cai et al., 2022).

Drug repurposing, a vital avenue for innovation in the pharmaceutical realm, offers an efficient approach to uncovering novel therapeutic applications. This is attributed to its shortened drug development cycle, cost-effectiveness, and well-established safety profiles. Trapidil (TRA), a captivating triazolopyrimidine compound, boasts a myriad of functionalities, encompassing vasodilation, anti-thrombosis, and enhancement of lipid metabolism (Nieder et al., 1995). It has been extensively utilized in clinical settings for patients with angina and hypertension. Moreover, TRA is employed in DM patients to optimize lipid metabolism (Nieder et al., 1995). Research has demonstrated that TRA can safeguard the ischemic heart from reperfusion injury by activating Protein kinase A II activity (Sichelschmidt et al., 2003). Notably, an increasing number of studies are beginning to highlight its anti-inflammatory and antioxidant potential, hinting that TRA may hold the key to unlocking new therapeutic horizons (Türck et al., 2018). The current research was undertaken to explore the therapeutic efficacy of TRA on DCM, its role in pyroptosis, as well as the underlying mechanisms of the oxidative-stress related pathway.

## 2 Materials and methods

### 2.1 Experimental model

The Animal Ethics Committee of Qiqihar Medical University approved all animal procedures and experiments under Protocol Number QMU-AECC-2021–237. Male C57BL/6 mice (n = 30, 6–8 weeks of age) were obtained from Qiqihar Medical University (Qiqihar, China). Throughout the study, the animals were housed in a controlled environment featuring a 12-h light/dark cycle and provided with standard mouse chow and water *ad libitum*. Following a 1-week acclimatization period, the mice were randomly allocated to the control group (CON) and the diabetic group (DT) in a 1:2 ratio. The DT group received streptozotocin (STZ, Solarbio, China) at a dose of 50 mg/kg, for 5 days in a row, while the CON group received an equal volume of sodium citrate buffer. Diabetes induction was confirmed by measuring tail vein blood glucose levels; a level of ≥16.7 mM indicated successful modeling of type I diabetes (Xu et al., 2024). Diabetic mice were randomly assigned to the model group (DCM), and the treatment group (DCM+TRA) at a 1:1 ratio. The DCM+TRA group received TRA (Shanghai Yuanye Bio-Technology, China) dosed at 25 mg/kg (Inaba et al., 1986; Kim and Kim, 2013), while the other groups received the same volume of 0.9% sodium chloride aqueous solution by oral gavage for 8 weeks, blood glucose levels were measured weekly, and the body weight of the mice was recorded. The recording was carried out continuously for 8 weeks. After the experiment was completed, the weekly body weights and blood glucose values of the mice in each group were counted and subjected to statistical analysis.

### 2.2 Echocardiography analysis

After 8 weeks of TRA treatment, mice were anesthetized with tribromoethanol (TIGERGENE, China; 0.2 mL/10 g). Their body temperature was sustained at approximately 37°C throughout the procedure. left ventricular end-diastolic diameter (LVIDd), left ventricular end-systolic diameter (LVIDs), ejection fraction (EF), and fractional shortening (FS) were measured using an echocardiography system (VINNO, China) (Liu et al., 2023).

### 2.3 Haematoxylin and eosin and Masson’s trichrome stainings of cardiac sections

Hearts were immersed in 4% paraformaldehyde for an overnight period at 4°C. Afterward, 5-µm paraffin sections were prepared. These sections underwent standard Haematoxylin and eosin (H&E) staining (Beyotime, China) as well as Masson’s trichrome staining (Solarbio, China). The stained sections were examined employing an inverted microscope (Nikon, China). The area of fibrosis was quantified with ImageJ software (Yan et al., 2017).

### 2.4 Transmission electron microscopy

Mice’s left ventricular heart tissue was collected and fixed in 2.5% glutaraldehyde in 0.1 M pH 7.3 sodium phosphate buffer, 4°C, overnight.The tissue was then exposed to 1% osmium tetroxide for 2 h. Next, it was stained with uranyl acetate dihydrate for an hour. Subsequently, the tissue was dehydrated with ethanol and then impregnated with epoxy resin. After slicing, staining was performed on ultrathin sections in the standard protocol using uranyl acetate and lead citrate. Ultra-structural analyses were conducted in a blind and unbiased manner using micrographs acquired by transmission electron microscopy (HITACHI, Japan) (Yaras et al., 2008).

### 2.5 Proteomics technology

Myocardial samples of each group were sent to Shanghai Yu Yue Biology for proteomic analysis. The steps were as follows: The appropriate protein solution was aliquoted into a 1.5 mL Eppendorf tube after quantification. Dithiothreitol (DTT) was introduced to finalize a concentration of 10 mM, followed by incubation of the mixture at 37°C for 1 h. Following this, iodoacetamide was introduced to achieve a final concentration of 50 mM, after which the reaction mixture was maintained in the dark at room temperature (RT) for a duration of 1 h. DTT was reintroduced to an ultimate concentration of 5 mM and incubated at RT for 10 min. The samples were then transferred to a 30 kDa ultrafiltration tube. After centrifugation, the collection liquid was removed, followed by the addition of 100 µL of 8 M urea to the ultrafiltration tube. This procedure was repeated twice. Next, 100 µL of NH4HCO3 at a concentration of 50 mM was introduced and centrifuged, and the collection liquid was removed. The process was performed three times in total. Subsequently, a fresh collection tube was employed, and trypsin was introduced into the ultrafiltration tube, maintaining a protein-to-enzyme ratio of 50:1. Enzymolysis was performed at 37°C for 16–20 h. The peptides were purified applying a C18 cartridge, followed by lyophilization and resuspension in 40 µL of a 0.1% formic acid solution. The solution’s concentration was determined using an OD280 reading. Peptide separation was performed using a high-performance liquid chromatography (HPLC) system with a nanoliter flow rate. Capillary HPLC was employed for separation, and analysis was conducted using an Orbitrap Fusion Lumos mass spectrometer (Thermo Scientific, USA).

### 2.6 Culture of neonatal mouse cardiomyocytes

Primary C57BL/6 neonatal mouse cardiomyocytes (NMCMs) were prepared by trypsin digestion and differential separation (Omatsu-Kanbe et al., 2018). Beating hearts were harvested from one-day-old newborn mice and instantly placed in pre-cooled Dulbecco’s Modified Eagle Medium (DMEM). The hearts were diced and digested using 0.25% trypsin at 4°C. The resulting digest was collected into DMEM culture medium with 10% fetal bovine serum (FBS) and then filtered through a 200-mesh stainless-steel screen. The filtrate was collected and centrifuged at 1,500 r/min for 5 min. The supernatant was removed, and the cell pellet was harvested. The cells were re-suspended in culture medium mentioned above, plated in cell culture dishes, incubating for 2 h in an incubator with the conditions of 37°C and 5% CO_2_. The non-adherent cells were replated by retrieving the culture medium, and 5-bromo-2′-deoxyuridine (BrdU) was added to inhibit the growth of non-cardiomyocytes (Sun et al., 2013; Zhang and Bao, 2021). The culture medium was refreshed after 48 h, and then the cells were cultured for an additional 24 h.

### 2.7 Cell viability

The methylthiazolyldiphenyl-tetrazolium bromide (MTT) assay was employed to evaluate the effect of TRA on NMCMs under high-glucose (HG) conditions (Leone and Engel, 2021). Cells were seeded in 96-well plates and incubated as above methods. Once the cells were confirmed to be in good condition, they were treated with TRA-containing medium at 37°C for 48 h. After that, 0.5 mg/mL MTT solution (Beyotime, China) took palce of the medium. The cells were incubated for 4 h under the conditions of 37°C and 5% CO_2_. Subsequently, the supernatant was removed, and the dark blue formazan crystals were dissolved in dimethyl sulfoxide (DMSO) (Kermel, China). Absorbance was measured at 570 nm by a microplate reader, and cell viability was computed,The dosage of TRA administered in each group is data obtained on the basis of a large number of preliminary experiments and subsequent formal experiments, according to the experimental method described in Section 2.7.

### 2.8 Treatment of neonatal mouse cardiomyocytes

NMCMs in good condition were randomly allocated to normal-glucose (NG), high-glucose (HG), and treatment groups (HG+TRA). NG group was cultured in a normal glucose medium at a concentration of 5.5 mmol/L. HG group was cultured in a high-glucose medium with a ultimate concentration of 33.0 mmol/L. The treatment group was cultured in the high-glucose medium and treated with 50 μmol/L TRA. All groups were cultured for 48 h for subsequent experiments.

### 2.9 ROS level analysis

NMCMs were seeded into a 24-well plate and treated as above method. After the treatment, the culture medium was removed. An appropriate amount of DCFH-DA was introduced to achieve a ultimate concentration of 10 μmol/L. The cells were then incubated in a 37°C cell incubator for 25 min in the dark. The NMCMs were rinsed three times with Phosphate Buffered Saline (PBS). A random field of view was observed applying a laser confocal microscope (ZEISS, Germany), imaged, and analyzed with ImageJ software (Sun et al., 2020).

### 2.10 Evaluation of mitochondrial membrane potential

As a fluorescent probe, 5,5′,6,6′-tetrachloro-1,1′,3,3′-tetraethylbenzimidazolylcarbocyanine (JC-1) shows different behaviors based on mitochondrial membrane potential (Δψm). At high Δψm, JC-1 forms J-aggregates within the mitochondrial matrix, showing red fluorescence. Conversely, when Δψm is low, JC-1 remains as a monomer in the mitochondrial matrix due to its inability to aggregate, emitting green fluorescence. The degree of mitochondrial depolarization can be evaluated by comparing the relative intensities of red and green fluorescence emissions (Yang et al., 2015). NMCMs were seeded into a 24-well plate and treated as above method. After treatment, the cells were rinsed using PBS. Then an appropriate volume of JC-1 working solution was introduced. After 15–20 min of incubation at 37°C and 5% CO_2_, removing the supernatant, the cells were rinsed twice using incubation buffer. Next, 1–2 mL of cell culture medium was added. Fields of view were randomly examined applying a laser confocal microscope (ZEISS, Germany), imaged, and analyzed using ImageJ software.

### 2.11 RNA extraction and quantitative real-time PCR

Total RNA was extracted from mouse myocardial tissue and NMCMs with TRIzol reagent (TaKaRa, Japan). First-strand cDNA was synthesized applying the PrimeScript™ RT Reagent Kit (TaKaRa, Japan). qPCR was performed using the TB Green^®^ Premix Ex Taq™ qPCR kit (TaKaRa, Japan). Primer sequences are listed in Table 1. Transcript levels were quantified using the relative quantitative method. Detected mRNA amounts were normalized to the endogenous control (β-actin). The relative value compared to the control sample was calculated using the 2^(-ΔΔCT) method.

**TABLE 1:** Primer sequences are given in the table.

Gene	Primer sequences
GPX3	F: 5′-ATCTACGAGTATGGAGCCCTTACC-3′ R: 5′-TTCAGTTCAAGGTATTGGTCTGTCAG-3′
β-Actin	F: 5′-TGTCACCAACTGGGACGATA-3′ R: 5′-GGGGTGTTGAAGGTCTCAAA-3′

### 2.12 siRNA transfection

Commercially available GPX3 and control siRNAs were obtained from Ruibiotech (China). NMCMs were seeded in a six-well plate, with 2 mL of sterile normal growth medium containing FBS added per well. Once the cells reached 60%–70% confluence, 100 pmol/L of control or targeted siRNA double-strands were transfected into the cells using Lipofectamine 2000 (Thermo Fisher, China). The cells were collected for subsequent experiments 48 h after transfection (Liu et al., 2018).

The cells were alocated into four groups: NG, GPX3 siRNA treatment (GPX3 siRNA), HG, and treatment (HG+TRA), as well as the TRA treatment plus GPX3 siRNA treatment (HG+TRA+GPX3 siRNA) group. Once the treatments were finished, cells were collected for Western blot analysis.

### 2.13 Western blot analysis

Cardiac tissue homogenates or NMCMs were lysed in Radio Immunoprecipitation Assay (RIPA) buffer (Beyotime, China) containing the protease inhibitor phenylmethylsulfonyl fluoride (PMSF). The protein lysates underwent centrifugation at a rotational speed of 13,500 rpm and a temperature of 4°C for a duration of 15 min. The concentration of total proteins present in the supernatant was measured through the utilization of a Bicinchoninic Acid (BCA) protein assay kit (Beyotime, China). Subsequently, the proteins were separated via sodium dodecyl sulfate-polyacrylamide gel electrophoresis (SDS-PAGE) and subsequently transferred onto a polyvinylidene difluoride (PVDF) membrane (Immobilon, USA). The membrane was then subjected to blocking with 5% skim milk powder under RT conditions for 1 hour. After that, it was incubated overnight at 4°C in the presence of primary antibodies. Post three washes with PBS, the membrane was incubated at RT for 1.5 h using secondary antibodies diluted at a ratio of 1:1,000. Visualization was performed using an enhanced chemiluminescence (ECL) luminescent solution (Meilunbio, China) and a luminescent image analyzer (Bio-Rad Laboratories, USA).

The primary antibodies used were: Cleaved-caspase1 (Med Chem Express, China); NLRP3, GPX3, COX2, Nrf2 and β-actin (Proteintech, China); Phospho-NF-κB, Cleaved-IL-1β (Affinity, China); Cleaved-IL-18 (ABclonal, China); GSDMD-N (Abcam, China). The secondary antibodies used were: Horseradish Peroxidase Labeled Goat Anti-Rabbit IgG (H+L) and Horseradish Peroxidase Labeled Goat Anti-Mouse IgG (H+L) (Beyotime, China).

### 2.14 Data analysis

The experimental data of each group were expressed as mean ± SEM (standard error of the mean). Data from Histochemical Staining, Fluorescence staining and Western blot were normalized to the control group and expressed as fold changes to account for technical variability across experiments (Wang et al., 2015). Statistical analyses were performed using SPSS 28.0 (IBM Corp). Normality was assessed via Shapiro-Wilk test (all datasets n ≤ 50). For normally distributed data with homogeneous variances (Levene’s test p ≥ 0.05), ordinary one-way ANOVA followed by Tukey’s post-hoc test was applied; if variances were unequal (p < 0.05), Welch’s ANOVA with Games-Howell post-hoc test was used; non-normal data were analyzed using Kruskal–Wallis test with Dunn-Bonferroni correction. Adjusted p-values <0.05 were considered significant.

## 3 Results

### 3.1 Trapidil ameliorates cardiac dysfunction and attenuates weight loss in DCM mice

**FIGURE 1:**
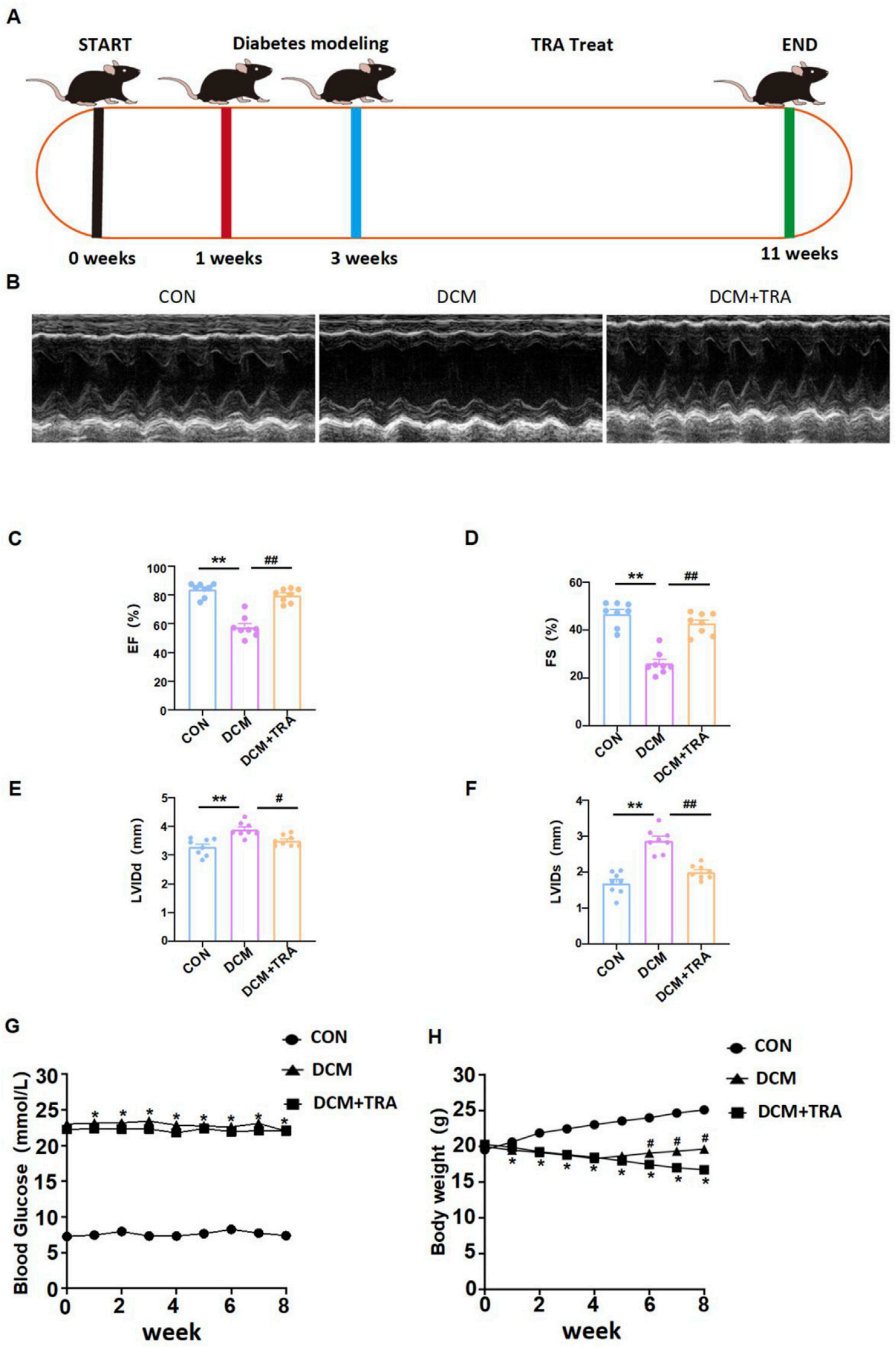
Trapidil ameliorates cardiac function in C57BL/6 diabetic cardiomyopathy mice. **(A)** Mouse modeling flow chart. **(B)** Representative M--mode echocardiography of mouse hearts in each group. **(C–F)** Quantitative analysis of LVIDd (mm), LVIDs (mm), EF (%) and FS (%). **(G, H)** Changes of body weight and blood glucose in each group. Data are presented as mean ± standard error, n = 8. CON: control group, DCM: model group, DCM+TRA: treatment group. The data passed the Shapiro-Wilk test, indicating a normal distribution (p > 0.05), except for the blood glucose data in the sixth week (p < 0.05). For the remaining data, the Levene’s test showed homogeneity of variances (p > 0.05). A ordinary one-way ANOVA analysis was performed, and the multiple comparisons were adjusted using the Tukey’s method. However, for the blood glucose data in the sixth week, Welch’s (p < 0.05) correction was applied. *p < 0.05, **p < 0.01, compared with the CON group; #p < 0.05, ##p < 0.01, compared with the DCM group.

Abnormal cardiac function is a primary clinical manifestation of DCM. In this study, we utilized a C57BL/6 mouse model induced with STZ to mimic DM, which closely replicates the common functional phenotype observed in the human diabetic heart. This model was employed to observe the impact of TRA on cardiac function in DCM (Figure 1A) (Ritchie and Abel, 2020). Echocardiographic assessments revealed that the LVIDd and LVIDs in the DCM group of C57BL/6 mice were markedly increased compared to the CON group. Furthermore, both EF (%) and FS (%) were markedly reduced in the DCM group. However, these parameters showed significant improvement following treatment with TRA (Figures 1B–F). These findings suggest that TRA effectively ameliorates cardiac dysfunction in DCM mice. Weekly measurements showed that DCM mice developed sustained hyperglycemia and progressive weight loss compared to controls (Figures 1G, H). TRA administration did not alter blood glucose levels (Figure 1G) but attenuated weight loss, with body weight recovery observed from the sixth week onward (Figure 1H). Collectively, these data demonstrate that TRA alleviates diabetic cardiomyopathy primarily by targeting cardiac remodeling rather than glycemic control.

### 3.2 Trapidil alleviates pathological injury of myocardial tissue in C57BL/6 DCM mice

**FIGURE 2:**
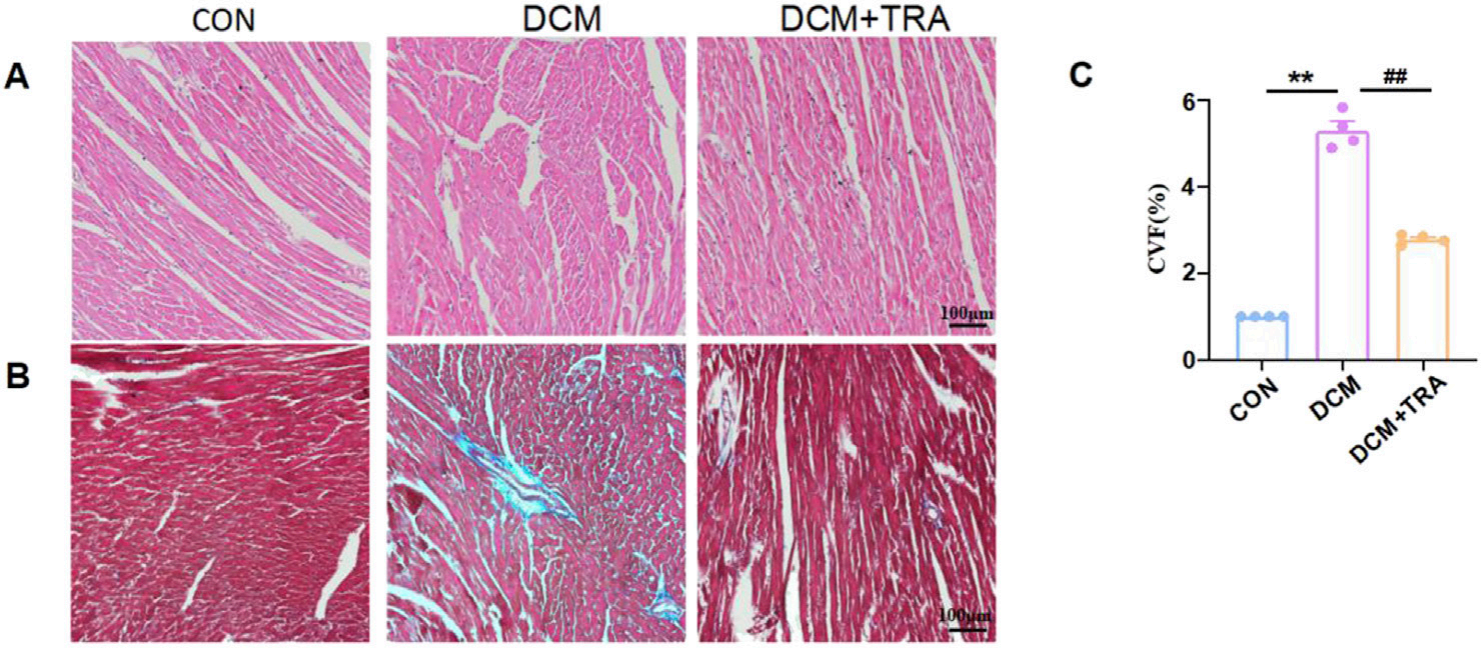
Trapidil ameliorates pathological injury of myocardial tissue in C57BL/6 diabetic cardiomyopathy mice. **(A)** H&E staining results of the left ventricle of mice from each group (scale bar, 100 µm). **(B)** Masson trichromatic staining results of the left ventricle of mice from each group (scale bar, 100 µm). **(C)** Quantitative statistical analysis of perivascular collagen volume fraction (CVF) in mice. Data are presented as mean ± standard error, n = 4. CON: control group, DCM: model group, DCM+TRA: treatment group. Since the data were normalized and there was at least one group of non-normal data, a non-parametric test was directly selected. Specifically, the Kruskal–Wallis test (p < 0.05) was used, and the post-hoc analysis was adjusted by the Dunn-Bonferroni method. **p < 0.01, compared with the CON group; ##p < 0.01, compared with the DCM group.

To ascertain the effects of TRA on the cardiac pathological changes in DCM, H&E staining and Masson’s trichrome staining were utilized to assess the myocardial histomorphology of mice across all groups. In the DCM group, cardiomyocytes exhibited varying sizes, disorganized arrangement, evident broken muscle fibers, and excessive collagen matrix accumulation. These abnormalities were significantly mitigated following TRA treatment (Figures 2A–C). These findings indicate that TRA effectively reverses myocardial cell damage in DCM mice.

### 3.3 Trapidil suppresses pyroptosis in myocardial tissue of C57BL/6 DCM mice

**FIGURE 3:**
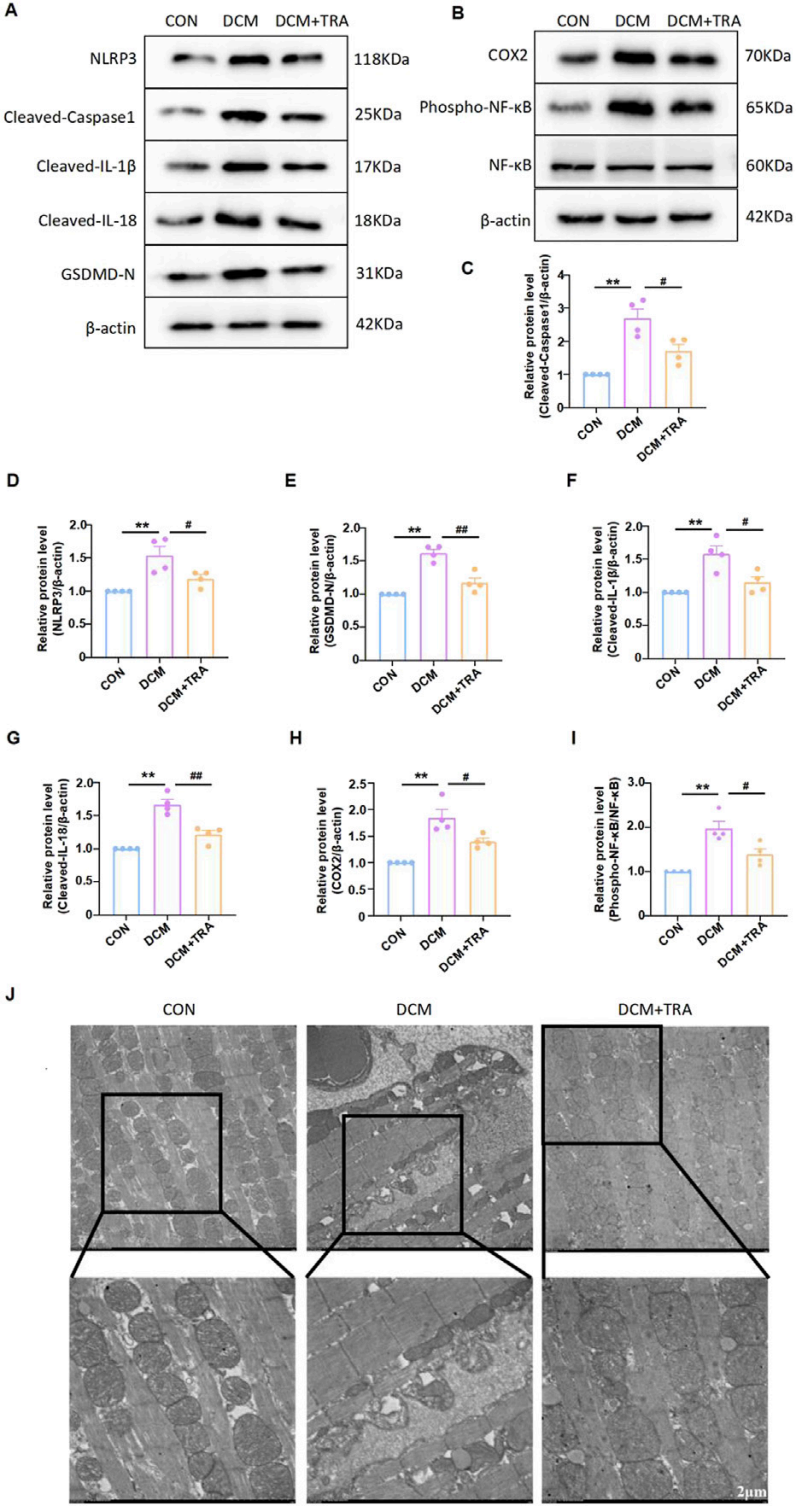
Trapidil ameliorates pyroptosis in myocardial tissue of C57BL/6 mice. **(A–I)** Representative Western blot images of cleaved-caspase1, NLRP3, phospho-NF-κB, COX2, Cleaved-IL-1β, Cleaved-IL-18, and GSDMD-N in the hearts of mice from each group. **(J)** Ultrastructure of the hearts of mice from each group observed by transmission electron microscopy (scale bar, 2 µm). Data are presented as mean ± standard error, n = 4. CON: control group, DCM: model group, DCM+TRA: treatment group.Since the data were normalized and there was at least one group of non-normal data, a non-parametric test was directly selected. Specifically, the Kruskal–Wallis test (p < 0.05) was used, and the post-hoc analysis was adjusted by the Dunn-Bonferroni method. **p < 0.01, compared with the CON group; #p < 0.05, ##p < 0.01, compared with the DCM group.

We explored whether TRA protects DCM mice by inhibiting pyroptosis in cardiac tissue. The expression of cleaved-caspase1, NLRP3, phospho-NF-κB, COX2, Cleaved-IL-1β, Cleaved-IL-18, and GSDMD in myocardial tissue of mice from each group was evaluated using Western blot. The results indicated that protein expression was aberrant in the DCM group, with significant improvements observed following TRA treatment (Figures 3A–I).

Transmission electron microscopy was employed to determine the effect of TRA on the ultrastructure of cardiomyocytes in DCM mice. The findings revealed mitochondrial swelling, cristae disarray, loss of intracellular contents, and the presence of pyroptosomes in cardiomyocytes of the DCM group. In contrast, after TRA treatment, the myocardial cells exhibited a uniformly shaped mitochondrial layer with abundant and well-organized cristae, situated between regularly arranged myofibrils, with no pyroptosomes observed (Figure 3J). These results suggest that pyroptosis occurs in cardiomyocytes of the DCM group and is significantly ameliorated following TRA treatment.

### 3.4 Trapidil inhibits high-glucose-induced pyroptosis of neonatal mouse cardiomyocytes

**FIGURE 4:**
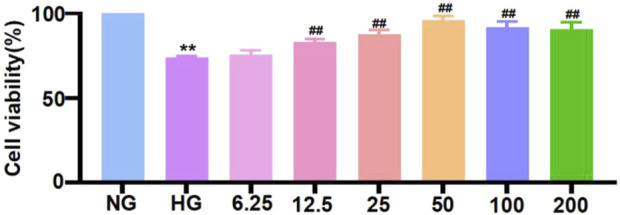
Cell viability of neonatal mouse cardiomyocytes cells in each group measured using methylthiazolyldiphenyl-tetrazolium bromide assay. The data are represented as mean ± standard error, n = 4. NG: normal glucose, HG: high glucose, HG+TRA: treatment group. Since the data were normalized and there was at least one group of non-normal data, a non-parametric test was directly selected. Specifically, the Kruskal–Wallis test (p < 0.05) was used, and the post-hoc analysis was adjusted by the Dunn-Bonferroni method. **p < 0.01, compared with the NG group; ##p < 0.01, compared with the HG group.

Before further verifying the inhibitory effect of TRA on pyroptosis at the cellular level, we used MTT assay to find the optimal concentration for TRA on NMCMs exposed to HG conditions. The results indicated that HG stimulation significantly decreased the viability of NMCMs. Starting at 12.5 μmol/L, various concentrations of TRA enhanced the viability of NMCMs in the HG environment. TRA reached its best protective effect at 50 μmol/L. When the concentration reached 100 μmol/L and 200 μmol/L, the protective effect of TRA on NMCMs in the HG environment did not further increase (Figure 4). In the present study 50 μmol/L was selected as the optimal concentration for TRA intervention in in vitro experiments.

**FIGURE 5:**
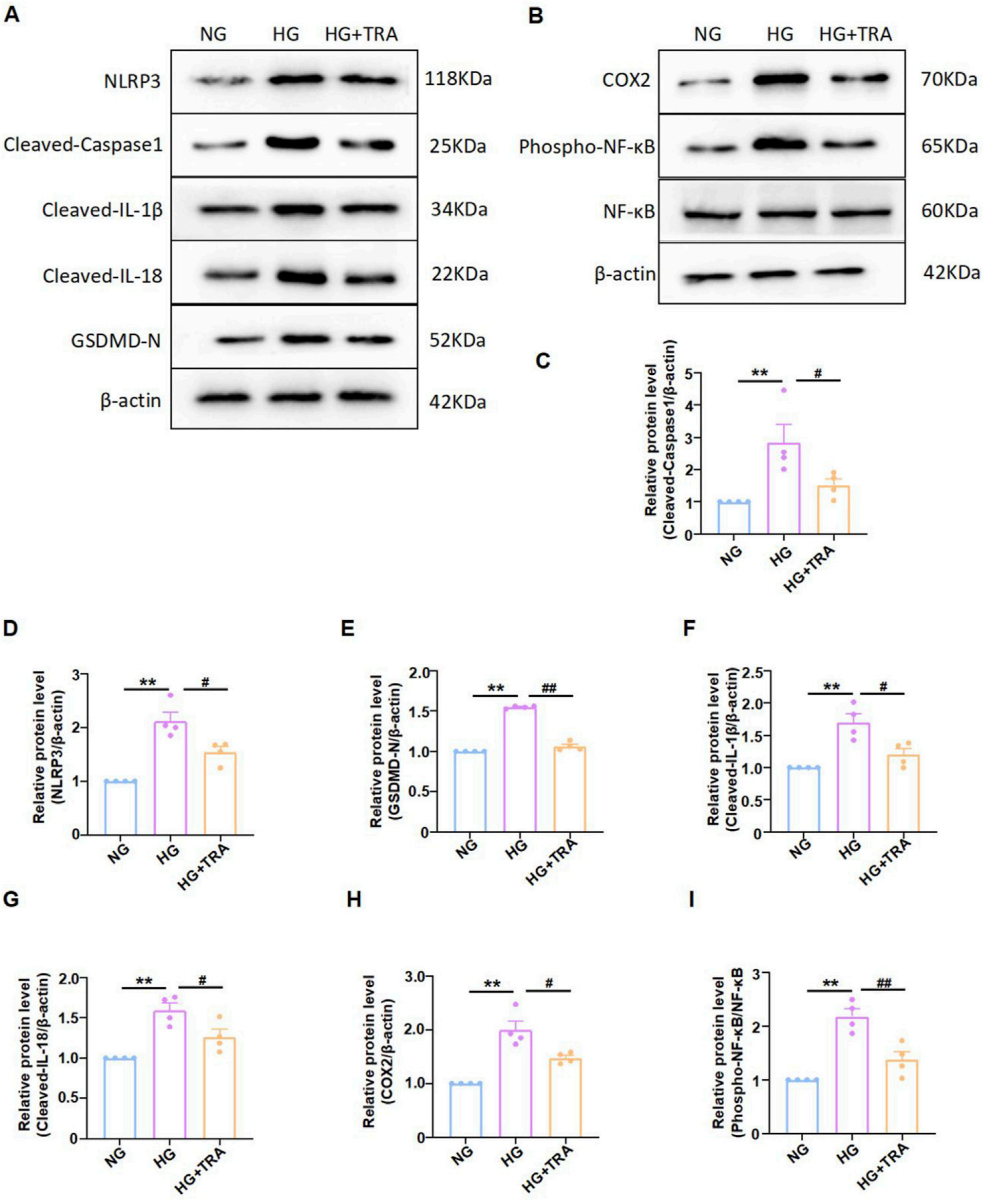
Trapidil inhibits pyroptosis in neonatal mouse cardiomyocytes cells. **(A–I)** Representative Western blot images of cleaved-caspase1, NLRP3, phospho-NF-κB, COX2, Cleaved-IL-1β, Cleaved-IL-18, and GSDMD-N in each group of NMCMs. Data are presented as mean ± standard error, n = 4. NG: normal glucose, HG: high glucose, HG+TRA: treatment group. Since the data were normalized and there was at least one group of non-normal data, a non-parametric test was directly selected. Specifically, the Kruskal–Wallis test (p < 0.05) was used, and the post - hoc analysis was adjusted by the Dunn-Bonferroni method. **p < 0.01, compared with the NG group; #p < 0.05, ##p < 0.01, compared with the HG group.

After determining the optimal concentration of TRA for NMCMs cells exposed to HG conditions, we further verified the inhibitory effect of TRA on pyroptosis at the cellular level. The results indicated that the effects of TRA on the expression of cleaved-caspase1, NLRP3, phospho-NF-κB, COX2, Cleaved-IL-1β, Cleaved-IL-18, and GSDMD in NMCMs cells in the HG environment were consistent with the findings from *in vivo* experiments (Figures 5A–H). These results indicated that TRA ameliorates pyroptosis in NMCMs cells under HG conditions, thereby confirming the protective effect of TRA against pyroptosis in DCM.

### 3.5 Trapidil inhibits oxidative stress in high-glucose-treated neonatal mouse cardiomyocytes

**FIGURE 6:**
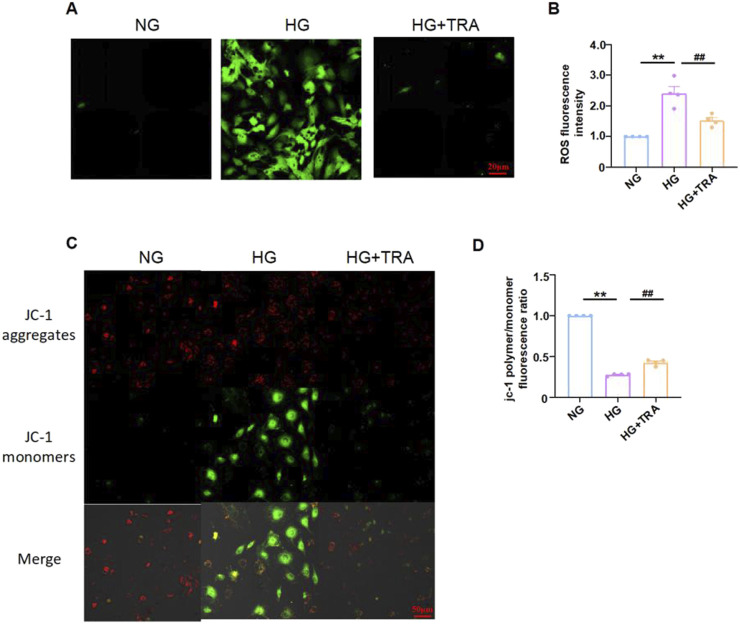
Trapidil inhibits oxidative stress occurring in neonatal mouse cardiomyocytes cells. **(A, B)** ROS levels in NMCMs of each group (scale bar, 20 µm). **(C, D)** Mitochondrial membrane potential levels in NMCMs of each group (scale bar, 50 µm). Data are presented as mean ± standard error, n = 4. NG: normal glucose, HG: high glucose, HG+TRA: treatment group. Since the data were normalized and there was at least one group of non-normal data, a non-parametric test was directly selected. Specifically, the Kruskal–Wallis test (p < 0.05) was used, and the post - hoc analysis was adjusted by the Dunn-Bonferroni method. **p < 0.01, compared with the NG group; ##p < 0.01, compared with the HG group.

In DM, hyperglycemia induces oxidative stress, with elevated ROS levels and reduced Δψm (Lingappan, 2018; Senoner and Dichtl, 2019). Oxidative stress is a key inducer of cell pyroptosis. To evaluate the impact of TRA on oxidative stress in NMCMs exposed to HG conditions, we performed ROS and JC-1 staining to evaluate ROS expression and mitochondrial integrity in each group. The results indicated that TRA significantly ameliorated the elevated ROS and reduced Δψm in NMCMs under HG conditions (Figures 6A–D). The findings suggest that TRA effectively inhibits oxidative stress in NMCMs in the HG environment.

### 3.6 Proteomic profiling of trapidil impact on cardiac tissue from diabetic cardiomyopathy mice

**FIGURE 7:**
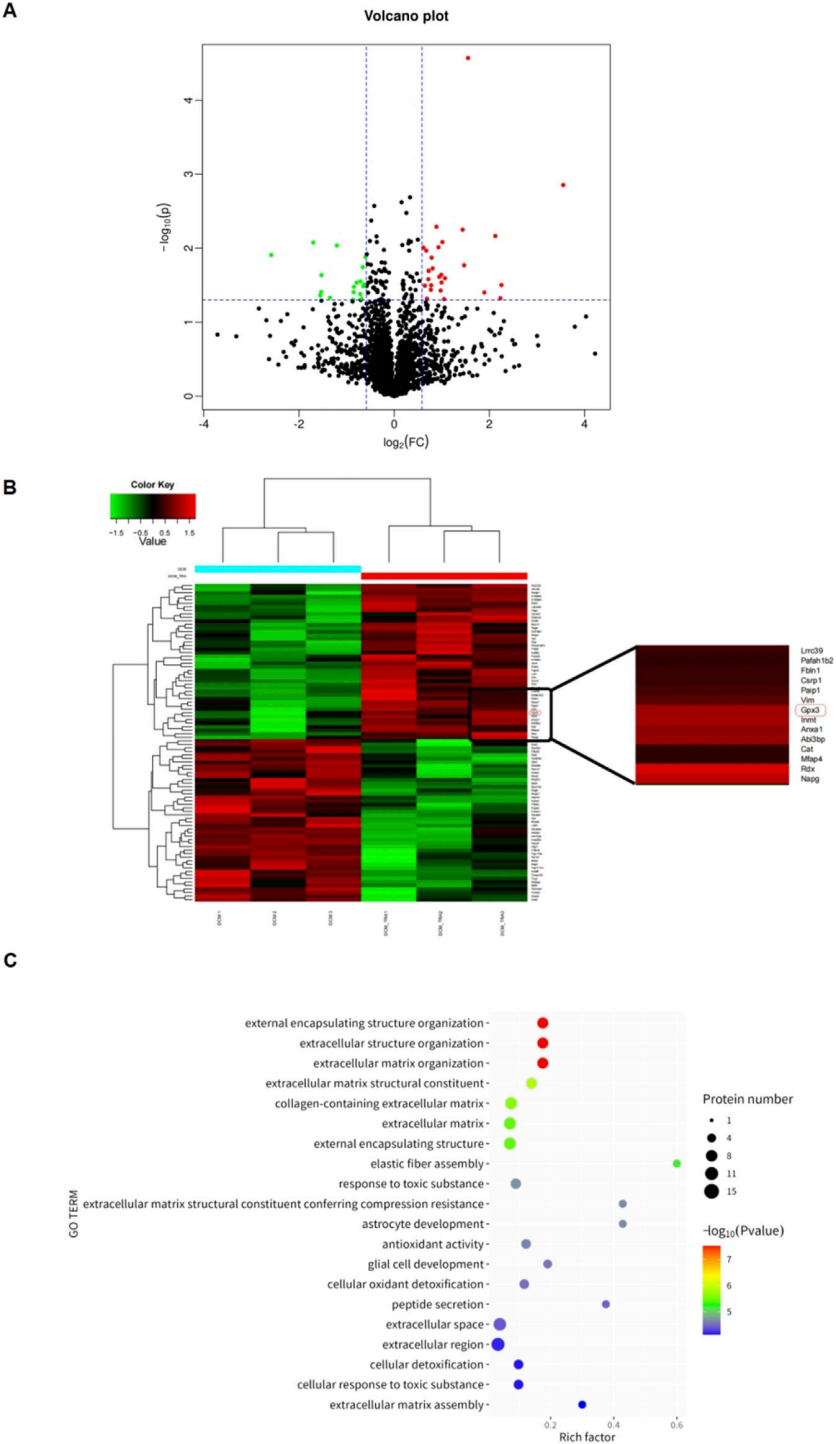
Proteomic analysis based on cardiac tissue from mice in each group. **(A)** Schematic diagram of volcanic plot results. **(B)** Cluster diagram of histone differences between DCM and DCM+TRA groups. **(C)** GO enrichment analysis results. n = 3. The p-value of the significance test was calculated using the T-test. Differential protein screening was performed under the condition of a 1.2-fold change (FC) and p < 0.05 threshold. Proteins with FC ≥ 1.2 and p < 0.05 were considered upregulated, and those with FC ≤ 0.833 and p < 0.05 were considered downregulated.

Proteomic analysis using mass spectrometry was conducted to uncover the targets by which TRA ameliorates DCM. The different proteins in DCM and DCM+TRA groups were screened, and a differentially expressed protein volcano map was calculated. The results identified 90 different proteins between DCM and DCM+TRA groups; 46 were upregulated, and 44 were downregulated. We concluded that these proteins may be key targets for TRA in treating DCM (Figure 7A).

Glutathione peroxidase 3 (GPX3), a secreted antioxidant enzyme, was among the proteins with differential expression. Compared with the DCM group, the protein expression of GPX3 was significantly upregulated in the DCM+TRA group (Figure 7B). GPX3, rich in the heart, liver, spleen, lung, etc., is essential for maintaining the normal physiological states of tissues and organs. It has been reported that the extracellular microenvironment with GPX3 deficiency can spontaneously induce the expression of NOX4 and the production of ROS in renal fibroblasts (Li et al., 2023a; b).

To elucidate the mechanistic involvement of GPX3 in mediating the therapeutic effects of TRA on DCM pathogenesis, we performed comprehensive functional enrichment analysis. As demonstrated in Figure 7C, GPX3 exhibits significant enrichment in redox-related biological processes. The enrichment results preliminarily identified that GPX3 is a key regulatory target for TRA in improving oxidative damage during the progression of DCM.

### 3.7 Trapidil upregulates the GPX3/Nrf2 pathway

**FIGURE 8:**
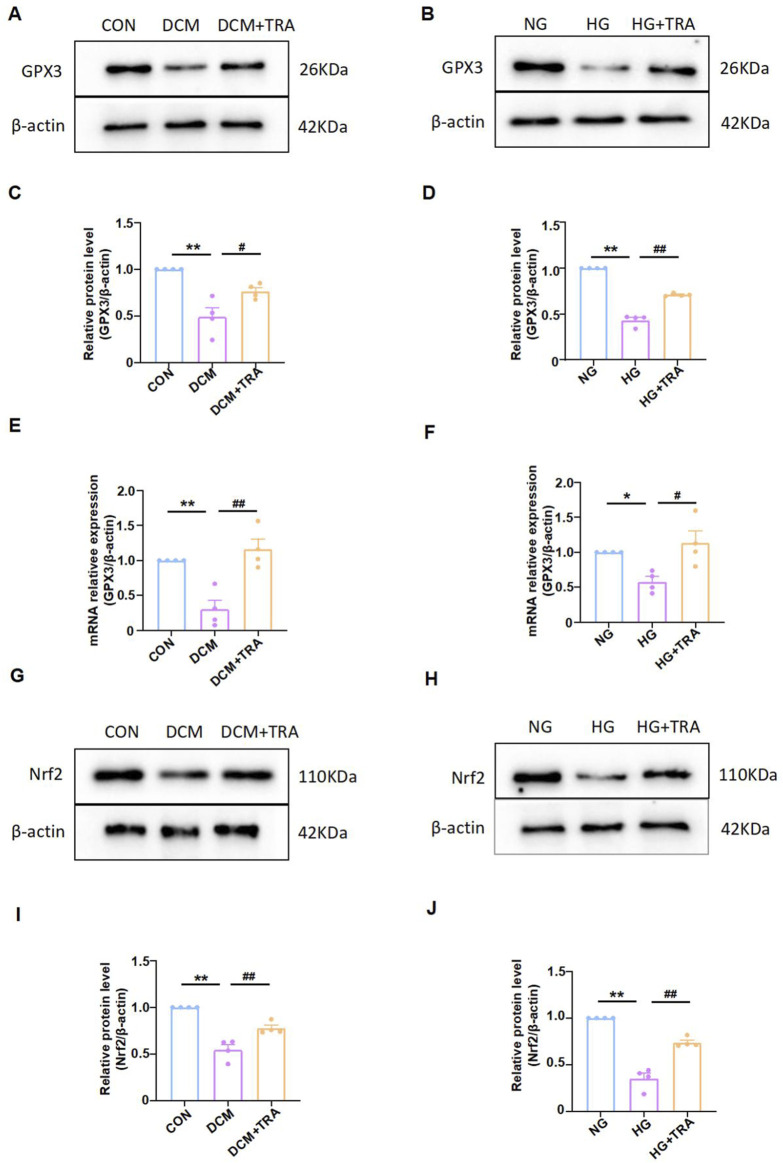
Trapidil upregulated the GPX3/Nrf2 pathway. **(A–D)** Representative Western blot images of GPX3 in each group. **(E, F)** mRNA expression of GPX3 in each group. **(G–J)** representative Western blot images of Nrf2 in each group. Data are presented as mean ± standard error, n = 4. CON and NG: control groups; normal glucose, DCM and HG: model groups; high glucose, DCM + TRA and HG + TRA: treatment groups. *p < 0.05, **p < 0.01, compared with the CON or NG groups; Since the data were normalized and there was at least one group of non-normal data, a non-parametric test was directly selected. Specifically, the Kruskal–Wallis test (p < 0.05) was used, and the post-hoc analysis was adjusted by the Dunn-Bonferroni method. #p < 0.05, ##p < 0.01, compared with the DCM or HG groups.

Based on the proteomic analysis, we measured the protein and mRNA levels of GPX3 in myocardial tissue and cardiomyocytes across all groups. Western blot indicated that GPX3 protein expression was markedly reduced in the model groups but was restored by TRA treatment (Figures 8A–D). Similarly, qPCR results indicated that GPX3 mRNA expression was decreased in the model groups and improved with TRA treatment (Figures 8E, F).

While GPX3 has been traditionally characterized as a secreted antioxidant enzyme, some fingdings demostrated it non-canonical intracellular role. In the hyperplasia model, GPX3 inhibits AMPK and upregulates Nrf2 in the prostate cells to improve benign prostatic hyperplasia (Li et al., 2023a; b). Nrf2 regulates NF-κB signaling (El-Shitany and Eid, 2019; Sajadimajd and Khazaei, 2018). It is emerging as a crucial regulator in cellular stress responses. We further analyzed the activated form of Nrf2 (molecular weight 95–110 kDa) (Lau et al., 2013) in myocardial tissue and cardiomyocytes across all groups applying Western blot. The results indicated that activated Nrf2 protein expression was markedly reduced in the model groups but was restored by TRA treatment (Figures 8G–J). The findings suggest that the GPX3/Nrf2 pathway may be a critical mechanism by which TRA inhibits myocardial pyroptosis.

### 3.8 Inhibition of GPX3 expression alters the effect of trapidil on pyroptosis and Nrf2 expression in neonatal mouse cardiomyocytes

**FIGURE 9:**
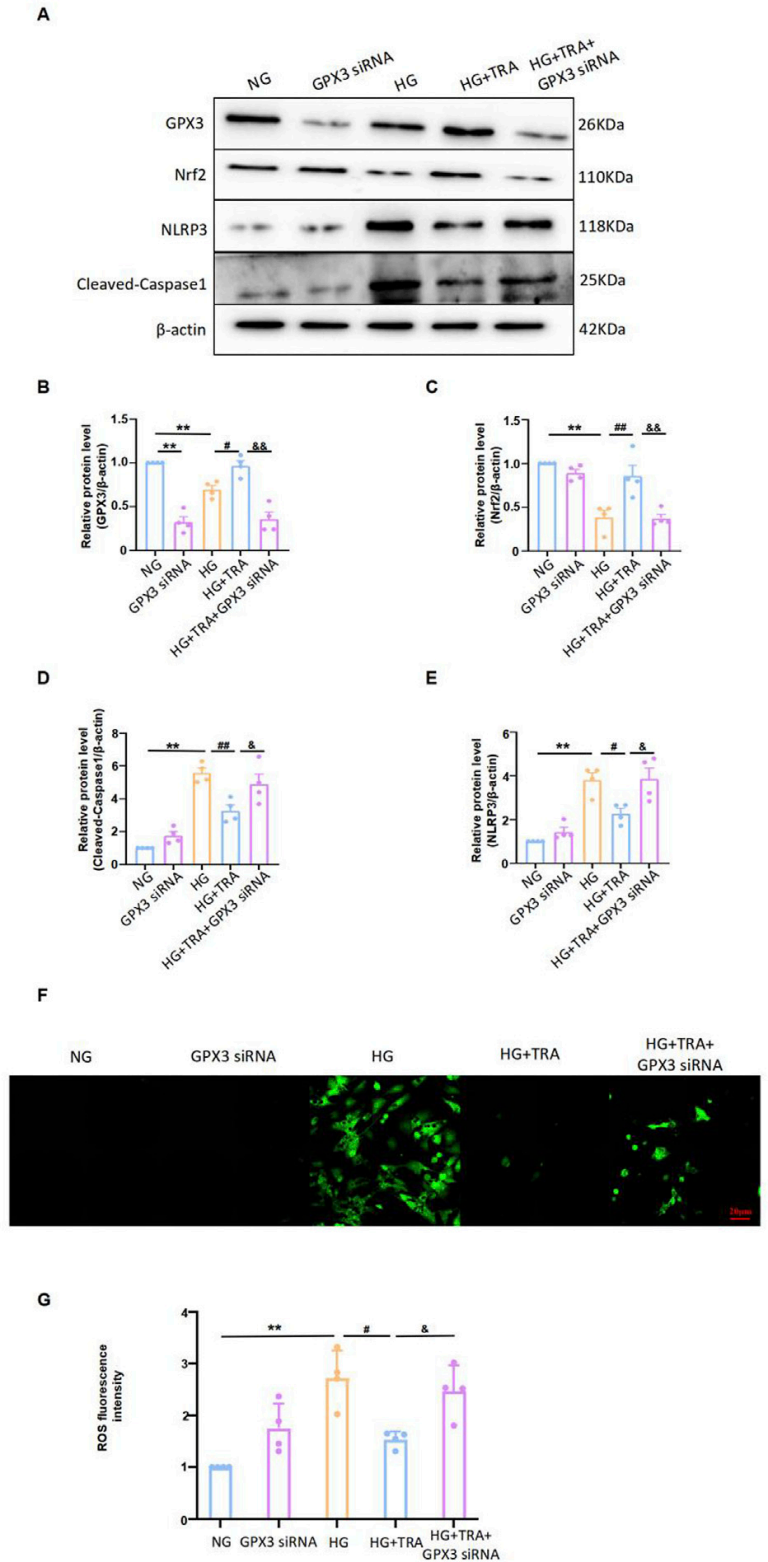
TRA inhibits pyroptosis in cardiomyocytes by regulating the GPX3/Nrf2 pathway. **(A–E)** Representative Western blot images of cleaved-caspase1, NLRP3, Nrf2, and GPX3 in NMCMs from each group; **(F, G)** The ROS fluorescence intensity in NMCM of each group. Data are presented as mean ± standard error, n = 4. NG: control group, GPX3 siRNA: GPX3 siRNA treatment group, HG: model group, HG+TRA: treatment group, HG+TRA+GPX3 siRNA: HG+TRA+GPX3 siRNA treatment group. **p < 0.01, compared with the NG group; Since the data were normalized and there was at least one group of non-normal data, a non-parametric test was directly selected. Specifically, the Kruskal–Wallis test (p < 0.05) was used, and the post-hoc analysis was adjusted by the Dunn-Bonferroni method. #p < 0.05, ##p < 0.01, compared with the HG group; &p < 0.05, &&p < 0.01, compared with the HG+TRA group.

We manipulated GPX3 expression to determine if the inhibitory effect of TRA on myocyte pyroptosis was affected. Results showed that after transfecting NMCMs with GPX3 siRNA to inhibit GPX3 expression (Figures 9A,B), the inhibitory effect of TRA on cleaved-caspase1 and NLRP3 levels in NMCMs under HG conditions was significantly reduced (Figures 9A,C–E). This indicates that inhibiting GPX3 expression reverses TRA’s protective effect against myocyte pyroptosis. Additionally, after GPX3 knockdown, the upregulation of Nrf2 expression by TRA was markedly decreased. Meanwhile, the inhibitory effect of TRA on ROS was also significantly weaken (Figures 9F,G). The findings suggest that GPX3 is a target protein of TRA in inhibiting pyroptosis, and the GPX3/Nrf2 pathway is crucial.

## 4 Discussion

As a prevalent complication of diabetes, DCM has increasingly become a key factor in the deterioration of the condition of diabetic patients in recent years. Its high incidence among the diabetic population and its significant impact on the progression of the disease cannot be ignored (Boudina and Abel, 2010). In 1972, Rubler first proposed the concept of DCM (Rubler et al., 1972). This milestone event paved the way for subsequent in-depth research in this area. Despite these efforts, DCM’s pathogenesis remains partially understood, and effective therapies are still absent. Current management largely relies on lifestyle modifications and classic hypoglycemic agents, such as metformin and glimepiride (Ritchie Abel, 2020). However, intensive glycemic control has not significantly reduced the risk of hospitalization or death from heart failure in diabetic patients (Zhan et al., 2022). Against this backdrop, our study provides a novel perspective by conclusively demonstrating that TRA exerts a cardioprotective effect on DCM through the inhibition of pyroptosis in cardiomyocytes via the GPX3/Nrf2 pathway. This finding not only highlights a potential therapeutic target but also opens new avenues for the treatment of DCM.

From the perspective of the pathogenesis, the hyperglycemia in DM will generate excessive ROS through the electron transport chain, which will then activate inflammasomes and trigger the pyroptosis process (Liu et al., 2024; Peng et al., 2022). Pyroptosis plays a central role in the development of DCM. It is accompanied by the release of a large number of pro-inflammatory factors. Once it progresses to the stage of severe tissue and cell damage, cell death will enter an irreversible state (Liu et al., 2024). Consistent with the metabolic hallmarks of diabetic cardiomyopathy (DCM), model mice exhibited sustained hyperglycemia, progressive weight loss, and characteristic cardiac pathology including cardiac dysfunction, myocardial fibrosis, and ultrastructural damage. TRA treatment attenuated weight loss and improved cardiac dysfunction without altering blood glucose levels, suggesting glucose-independent cardioprotective effects. Mechanistically, DCM progression was associated with upregulated pyroptotic markers: myocardial tissues and HG-treated NMCMs showed elevated expression of cleaved caspase-1, NLRP3, phospho-NF-κB, and GSDMD compared to controls, accompanied by increased pyroptosome formation. TRA administration normalized these perturbations, reducing inflammatory cytokines including COX2, Cleaved-IL-1β, Cleaved-IL-18. Furthermore, this study identified elevated ROS levels and reduced mitochondrial membrane potential in NMCMs under HG conditions, which were significantly ameliorated by TRA treatment. Collectively, these findings indicate that both NMCMs under HG conditions and DCM mice are deeply entangled in the predicament of pyroptosis while TRA, acting as a “savior,” significantly inhibits the pyroptosis process.

GPX3, a glutathione peroxidase family member, is also known as selenium-dependent glutathione peroxidase. It catalyzes the reaction between glutathione and hydrogen peroxide or other peroxides, thereby decomposing peroxides and ROS and neutralizing oxygen free radicals. This process effectively eliminates these harmful substances and protects cells from oxidative damage (Wang and He, 2022). In the renal cell line (Caki-2), hypoxia significantly upregulated GPX3 promoter activity, highlighting its role as a critical protective component against acute hypobaric hypoxia in rats (Bierl et al., 2004; Burk et al., 2011). Various ROS inducers have been shown to stimulate GPX3 mRNA expression in lung epithelial cells (Rahman et al., 1998). This study revealed for the first time that GPX3 protein and mRNA levels were considerably reduced in the myocardial tissue of DCM mice and in NMCMs treated with HG. Specifically, HG exposure inhibited GPX3 protein and mRNA expression in cardiomyocytes.

While GPX3 has been traditionally characterized as a secreted antioxidant enzyme, some fingdings demostrated it non-canonical intracellular role. In the hyperplasia model, GPX3 inhibits AMPK and upregulates Nrf2 in the prostate cells to improve benign prostatic hyperplasia (Li et al., 2023a; Li et al., 2023b). Nrf2 is a primary regulator of cellular redox and metabolic homeostasis. Nrf2 is sequestered by cytoplasmic Keap1 and targeted for proteasomal degradation under basal conditions. Once dissociates from Keap1 and translocates to the nucleus, it exerts antioxidant and anti-inflammatory effects. Specifically, it regulates ROS expression levels (Rahman et al., 1998) and negatively regulates the NF-κB signaling pathway (Wang et al., 2022a; Wang et al., 2022b). While some evidence indicates that oxidative stress typically activates Nrf2 through Keap1 oxidation-induced nuclear translocation (Bellezza et al., 2018), our study revealed a paradoxical suppression of Nrf2 signaling in diabetic cardiomyopathy, with HG inhibiting Nrf2 protein expression in cardiomyocytes. This suggests chronic hyperglycemia may override canonical oxidative stress responses, potentially through KEAP1-independent degradation pathways or epigenetic silencing of Nrf2 transcription.

This study, through multiple analyses, verified that TRA upregulated GPX3 protein and mRNA and Nrf2 protein expression in myocardial tissue of DCM mice and HG-treated NMCMs. Remarkably, after knocking down the expression of GPX3 mRNA using the transient transfection siRNA technique, the inhibitory effect of TRA on cleaved caspase1 and NLRP3 pyroptosis signature proteins significantly weakened. Concurrently, the expression of Nrf2 protein was also significantly decreased. These crucial data strongly indicate that GPX3 is the core target for TRA to inhibit DCM pyroptosis, and the GPX3/Nrf2 pathway constitutes a critically important signaling pathway.

Results above also accentuate the role of GPX3 and Nrf2 in DCM’s pathogenesis. This finding suggests that GPX3/Nrf2 downregulation may make contribution to the oxidative stress, pyroptosis and subsequent cardiac dysfunction observed in DCM. It is summarized that several signaling pathways currently participate in pyroptosis in DCM, including TLR4/NF-kB (Wang F. et al., 2019), AMPK/ROS/TXNIP (Wang et al., 2022a; Wang et al., 2022b) and AMPK/SIRT1/Nrf2/HO-1/NF-kB (Wang Y. et al., 2019) inflammasome signaling pathways, among others. In the present study, we propose that GPX3/Nrf2/NF-kB NLRP3 inflammasome signaling pathways is also involved in pyroptosis in DCM.

TRA is a multifunctional agent characterized by its potent coronary vasodilatory effects, antithrombotic properties, and ability to regulate lipid metabolism in diabetic patients (Nieder et al., 1995). Despite these attributes, the therapeutic potential of TRA in DCM has not been comprehensively investigated. Systematic exploration of TRA’s application and underlying mechanisms in DCM treatment is of significant importance. Such research can not only reduce the costs and time associated with the development of novel DCM therapies but also markedly enhance the success rate of such endeavors, thereby offering renewed hope to patients severely affected by DCM.

**FIGURE 10:**
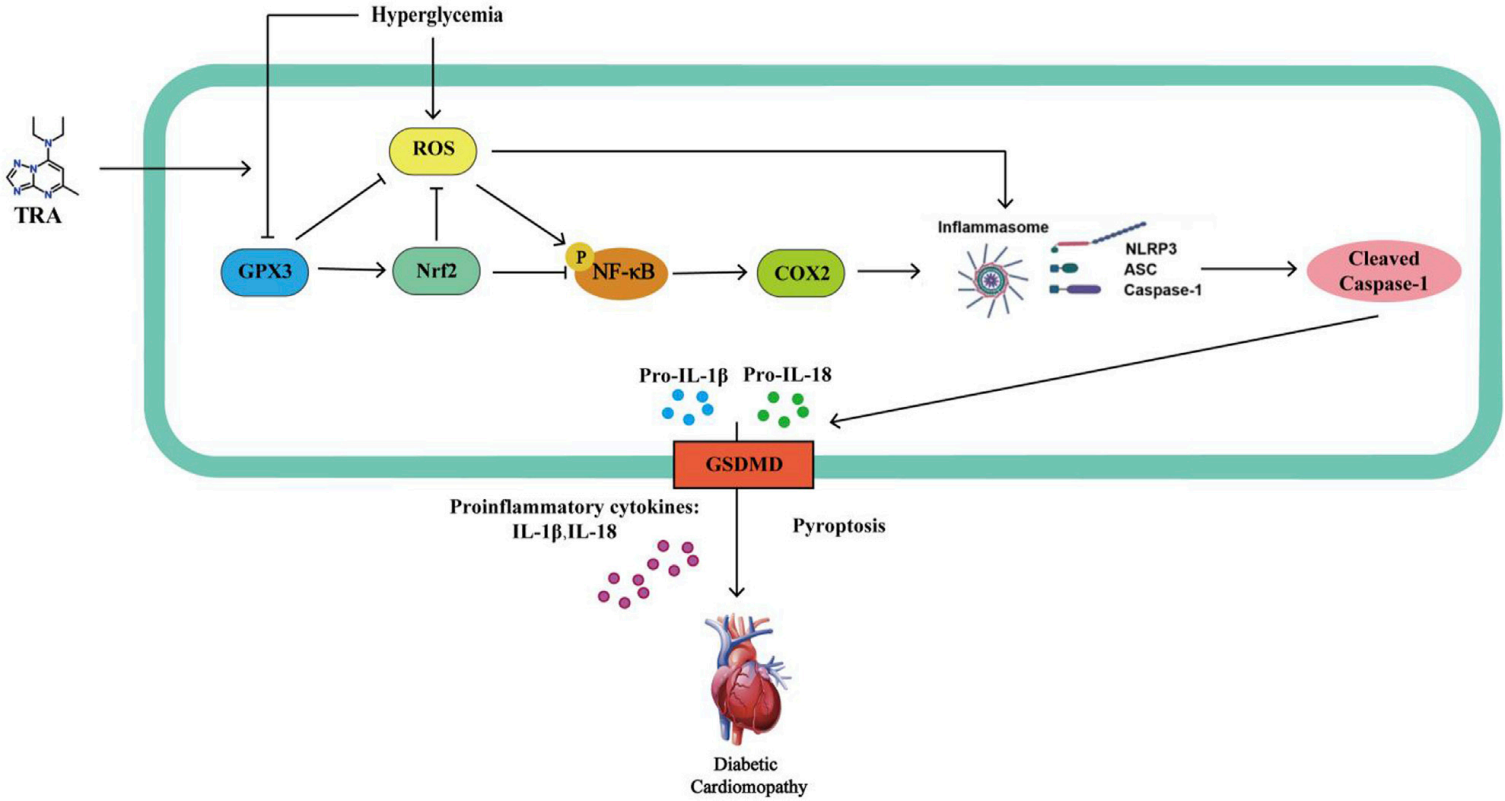
Trapidil inhibits myocardial pyroptosis in DCM via the GPX3/Nrf2 pathway. *In vivo* and *in vitro* studies revealed that TRA activates the expression of GPX3 and Nrf2, thereby decreasing the levels of downstream signaling molecules, including phospho-NF-κB, COX2, NLRP3, cleaved-caspase1, IL-1β, IL-18, and GSDMD. This cascade of effects aids in protecting cardiac function and maintaining mitochondrial homeostasis, while alleviating oxidative stress and pyroptosis in cardiomyocytes. TRA: Trapidil; GPX3: glutathione peroxidase 3; Nrf2: nuclear factor E2 related factor 2; ROS: Oxidative stress; NF-κB: nuclear factor-κB; COX2: cyclooxygenase 2; NLRP3: Nucleic acid-bound oligomerized domain-like receptor protein 3; Caspase1: aspartate proteolytic enzyme 1; ASC: GSDMD: gasdermin D.

In summary, this study has obtained notable results. TRA has shown an improvement in the myocardial tissue pathology, cellular ultrastructure, and cardiac function of DCM mice. It suppresses the pyroptosis process in the myocardial tissues of DCM mice and HG-treated NMCMs, and regulates the GPX3/Nrf2/NF-κB/cleaved-caspase1 pathway. These effects contribute to curbing the development of DCM. This study demonstrates that TRA, a traditional medication for coronary heart disease, holds potential for the treatment of DCM. TRA enhances Nrf2 signaling through a GPX3-dependent mechanism. Moreover, this study proposes that the GPX3/Nrf2 pathway may serve as a target for inhibiting pyroptosis, offering a novel therapeutic perspective. This could potentially offer solutions for the prevention and treatment of DCM (Figure 10).

While our data establish GPX3 as a necessary mediator of TRA-induced Nrf2 activation, the precise molecular initiating event—whether TRA directly engages GPX3 transcription/translation machinery or acts through intermediate signaling hubs—remains to be fully elucidated. This mechanistic ambiguity represents an important frontier for future translational studies on TRA’s pleiotropic cardioprotective effects. Beyond GPX3, intracellular isoforms (GPX1/2/4) may orchestrate compensatory antioxidant responses. For instance, GPX3 overexpression upregulates GPX4 in prostate tissue (Li et al., 2023a; b). Our exclusive focus on GPX3 precludes a holistic understanding of this family’s crosstalk, a gap addressable through GPX1/4 knockout models and interactome mapping. While the current study focuses on elucidating TRA’s cardioprotective effects through modulation of pyroptosis and the GPX3/Nrf2 axis, future work will systematically explore its regulatory roles in lipid metabolism and insulin signaling-two central pathogenic drivers of diabetic cardiomyopathy.

The therapeutic potential of TRA warrants careful consideration of its pharmacokinetic and clinical translation profile. Studies have shown that the clinical therapeutic dose of TRA is 100 mg 3 times a day (Harder et al., 1996). Our selected *in vivo* dose (25 mg/kg/day) represents a conservative 58% reduction compared to the clinical equivalent dose (60 mg/kg) derived from body surface area normalization (Km coefficients), suggesting a favorable safety margin for human applications. Notably, the *in vitro* concentration of TRA (50 μmol/L) is 26-fold lower than concentrations previously shown to modulate mitogen-activated protein kinase cascades (1.3 mmol/L) (Hoshiya Awazu, 1998), underscoring its selective action within a safe therapeutic window.

However, clinical translation must account for TRA’s hepatic clearance dynamics. Pharmacokinetic studies indicate that TRA’s elimination is highly sensitive to hepatic impairment (Berndt et al., 1996), necessitating dose adjustments in cirrhotic patients. While our DCM model did not explore liver comorbidities, future investigations should integrate population pharmacokinetic modeling to optimize dosing regimens for patients with concurrent metabolic and hepatic disorders.

It is worth noting that the study’s duration was relatively short, leaving the long-term effects on cardiac function and pyroptosis in DCM incompletely explored. Relying solely on animal and *in vitro* models, which fail to fully mirror human complexity due to the lack of human sample research, makes the extrapolation of conclusions to humans uncertain. To bridge this gap, clinical trials involving human subjects are essential to validate these findings and assess their applicability. Future studies should address these limitations by conducting human clinical trials, recruiting patients with DCM of diverse ages and disease severities to verify the drug’s effectiveness. Additionally, extending the study duration and following up patients for several years will clarify the impact of long-term TRA use on cardiac function and overall health, thus optimizing its therapeutic benefits and ensuring safe and effective clinical application.

## Data Availability

The original contributions presented in the study are publicly available. This data can be found here: www.uniprot.org, accession number UP000000589.
